# Recommendations on postoperative strain and physical labor after abdominal and hernia surgery: an expert survey of attendants of the 41st EHS Annual International Congress of the European Hernia Society

**DOI:** 10.1007/s10029-021-02377-w

**Published:** 2021-02-24

**Authors:** S. Schaaf, A. Willms, R. Schwab, C. Güsgen

**Affiliations:** Department of General, Visceral and Thoracic Surgery, German Armed Forces Central Hospital Koblenz, Rübenacher Str. 170, 56072 Koblenz, Germany

**Keywords:** Abdominal surgery, Hernia surgery, Postoperative strain, Incisional hernia

## Abstract

**Background:**

There are no valid recommendations or reliable guidelines available to guide patients how long they should refrain from lifting weights or returning to heavy physical labor after abdominal or hernia surgery. Recent studies found that surgeons’ recommendations not to be evidence-based and might be too restrictive considering data on fascial healing and incisional hernia development. It is likely that this impairs the patient’s quality of life and leads to remarkable socio-economic costs. Hence, we conducted this survey to gather international expert’s opinions on this topic.

**Materials and methods:**

At the 41st Annual International Congress of the EHS, attending international experts were asked to complete a questionnaire concerning recommendations on given proposals for postoperative refrain from heavy work or lifting after abdominal surgery and also after hernia repairs.

**Results:**

In total, 127 experts took part in the survey. 83.9% were consultants with a mean experience since specialization of more than 11 years. Two weeks of no heavy physical strain after laparoscopic surgery were considered sufficient by more than 50% of the participants. For laparotomy, more than 50% rated 4 weeks appropriate. For mesh-augmented sublay and IPOM repair of ventral or incisional hernias, more than 50% rated 4 weeks of rest appropriate. For complex hernia repair, 37% rated 4 weeks reasonable. Two weeks after, groin hernia surgery was considered sufficient by more than 50% of the participants.

**Conclusion:**

Following groin hernia repair (Lichtenstein/endoscopic technique) and laparoscopic operation, the majority agreed on the proposal of 2 weeks refraining from physical strain. Four weeks of no physical strain were considered appropriate by a majority after laparotomy and open incisional hernia repair. However, the results showed substantial variation in the ratings, which indicates uncertainty even in this selected cohort of hernia surgery experts and emphasizes the need for further scientific evaluation. This is particularly remarkable, because a lack of evidence that early postoperative strain leads to higher incisional hernia rates.

**Trial registration:**

Number DRKS00023887.

## Introduction

Surgical procedures underwent a technical development in the recent three decades. Due to the further development of minimally invasive techniques and implementation of enhanced recovery after surgery protocols (ERAS), length of hospital stay decreased and early mobilization became an essential element in the postoperative course [1].

However, the occurrence or recurrence of incisional hernias after abdominal or hernia surgery is a common complication, as they are reported in 12.8% within the first 2 years of abdominal surgery. [2] A negative impact of early postoperative physical strain, that might lead to fascial tearing or trauma and, hence, to high incisional hernia rates is widely suspected [3].

That might be reflected in the postoperative recommendations given to patients. Important elements of postoperative management are those recommendations of convalescence, especially regarding physical activity and return to work. The advice given to patients by their surgeons directly affects the patients’ behavior, their participation in daily and social life and, of course, the duration of sick leave and return to work. How long patients should refrain from lifting weights or returning to regular physical activity after abdominal or hernia surgery is debated and data from hernia research emphasize a major impact of collagen metabolism in the pathogenesis of incisional or primary ventral hernias [4, 5].

Study data from Germany prove a considerable variance of recommendations given to patients [5]. Moreover, such proposals are not evidence-based and might be too restrictive, considering the available data on fascial healing and incisional hernia development.

For inguinal hernia repair, it could be shown that there is an extensive database, and there is no evidence which justifies restrictive recommendations [6, 7]. Also, even no restriction or only a few days is not associated with higher rates of complications or recurrences. Hence, the International Guidelines for Groin Hernia Management emphasized that patients should be encouraged to return to their normal activities as soon as possible (upgrade to strong recommendation). [8]

Such a conclusive database does not exist for abdominal or incisional/ventral hernia surgery and thus, guidance of patients in the postoperative period cannot be validated by scientific results, yet. To further evaluate this unanswered question, we conducted this survey to gather international expert’s opinions on this topic. The results have to be interpreted with regard of being low level of evidence: level D according to GRADE guidelines [9] as the GRADE guidelines might be of more widespread use compared to Hadorn or Oxford classification. Expert panel statements might be biased, thus the given recommendations are not binding.

## Materials and methods

At the 41st Annual International Congress of the EHS 11—14 September 2019 in Hamburg, attending experts were asked to complete a questionnaire. We included only active active surgeons with a self-reported special interest (i.e., active research) and/or superior experience in that field. The only personal data collected was the profession (resident/consultant) and the duration since graduation/specialization. We asked for recommendations on given proposals for postoperative refrain from heavy work or lifting.

**Fig. 1:**
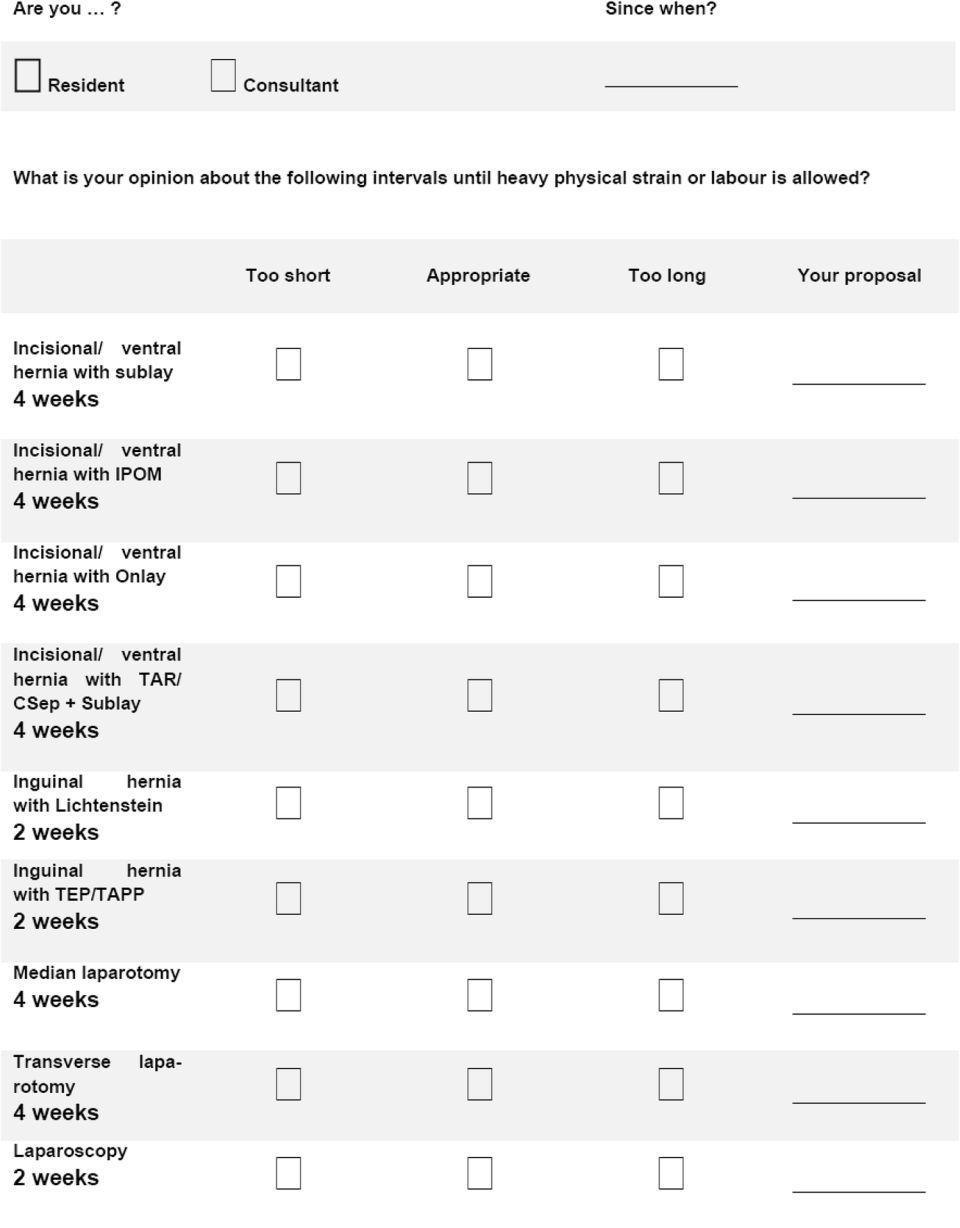
Depiction of the questionnaire used for the survey

Four weeks of refrain from heavy work and activity were given interval as proposal for abdominal surgery with vertical midline or transverse laparotomy and 2 weeks for laparoscopic surgery. Four weeks were the given interval as a proposal for incisional hernia repair (sublay, IPOM (intraperitoneal onlay mesh), onlay or complex repair). Complex repair was considered techniques such as transversus abdominis release or component separation techniques. Two weeks were proposed for groin hernia repair (Lichtenstein or endoscopic repair with TEP/TAPP). The proposals were to be qualified too short, too long, or appropriate. If not suitable, the experts were asked to give their recommendations (Fig. 1). This study was intended to add some evidence in terms of evaluating the acceptance of the given proposals. Those were based on the conclusions drawn from a German hospital survey and literature review on postoperative refrain from heavy work and activity [5].

The participation of experts was voluntary, and no benefits were offered. Also, no personal data were recorded in addition to the answers given in the questionnaire.

After the end of the survey, the data were recorded and descriptively analyzed with Excel (vs. 2016, Microsoft, Redmont, Washington, USA) and SPSS (vs. 20, IBM, Armonk, New York, USA). Differences between the ratings were tested for significance with the Chi-squared test. The level of significance was set to *p* = 0.05.

## Results

In total, 127 experts took part in the survey. 83.9% were consultants with a mean experience since specialization of about 11 years. The remaining 16.1% were residents and reported nearly 4 years since graduation. The following results did not differ between the subgroups of the residents and consultants; therefore, we report the groups together.

### Abdominal surgery

The given proposal was 4 weeks for vertical midline and transverse laparotomy, which was found to be appropriate in 56.7/52.8%, too short in 31.5/29.9% (recommendation 6–12 weeks), and too long in 11.8% in both cases (recommendation 1–3 weeks).

Two weeks were proposed for laparoscopy, which was rated appropriate in 57.5%, in only 4.7% as too short (recommendation 4 weeks), and 36.2% as too long (recommendation 0–1.5 weeks). These differences were statistically significant (*p* = 0.000; Chi-squared test).

**Fig. 2:**
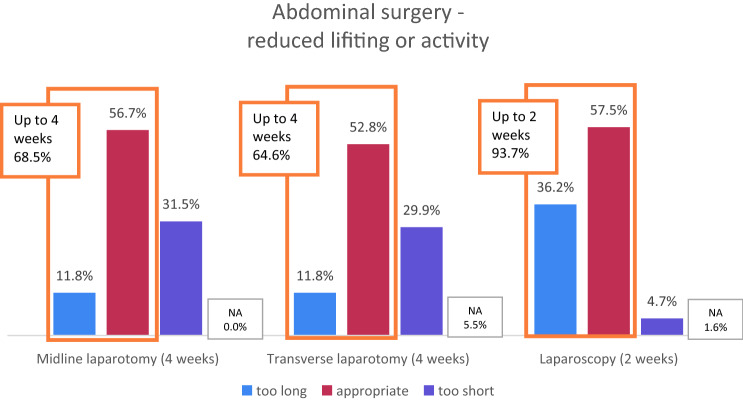
Ratings of 4 weeks for open abdominal surgery (midline or transverse laparotomy) and 2 weeks for laparoscopy. *NA* not answered

Almost 70% considered an interval of up to 4 weeks sufficient after open abdominal surgery (midline and transverse incisions). The ratings were even more straightforward for laparoscopy, as more than 90% recommended 2 weeks or shorter of reduced physical activity or lifting after laparoscopic surgery (Fig. 2).

### Groin hernia repair

For groin hernia repair, the given proposal was 2 weeks. That was rated appropriate in 55.9% for open repair (Lichtenstein) or 59.8% for endoscopic repair (TEP/TAPP). It was ranked too short in 31.5/11.0% (Lichtenstein/endoscopic) and too long in 11.8/28.3% (Lichtenstein/endoscopic TEP-TAPP). These differences were statistically significant (*p* = 0.000; Chi-squared test). The proposed intervals were either 0–1 weeks for those who rated 2 weeks too long and 3–16 weeks for those who considered 2 weeks too short.

**Fig. 3:**
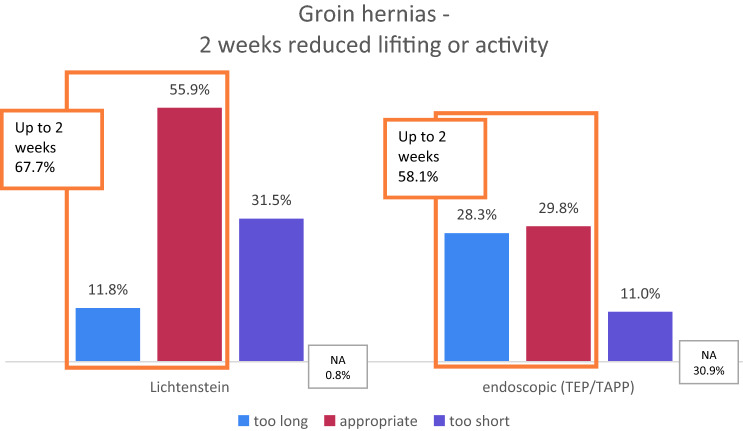
Ratings of 2 weeks reduced physical activity after groin hernia surgery. *NA* not answered

It was found that the majority of 58–68% considered up to 2 weeks of reduced physical activity or heavy lifting after a groin hernia repair to be sufficient (Fig. 3). A remarkable proportion of almost one third (28%) rated 2 weeks even too long after endoscopic groin hernia repair (Fig. 3).

### Incisional/ventral hernia repair

The given interval was 4 weeks for hernia repair. The results are shown in Table 1. For sublay, 55.1% rated it appropriate, while it was too short for 31.5% and too long for 13.4%. Those who rated too long recommended intervals of 2–3 weeks, and those who rated too short suggested 5–12 weeks with a mean of 7 weeks.

**Table 1:** Overview of survey results. The right column shows the means and standard deviations (ranges) of the individual proposals by the participants for refrain from lifting or heavy physical labor after the surgical procedures if they did not rate the given proposal of four/two weeks appropriate. IPOM, intraperitoneal onlay mesh.

Resident	18 (16.1%)	
Consultant	102 (83.9%)	
Time (years) since		
Graduation (resident)	4.3 ± 3.7 (1–15)	
Specialization (consultant)	11.3 ± 8.9 (1–40)	
Incisional/ventral hernia repair		
Sublay—4 weeks		Proposal
Too short	40 (31.5%)	7.0 ± 1.7 (5–12)
Appropriate	70 (55.1%)	
Too long	17 (13.4%)	2.3 ± 0.5 (2–3)
IPOM—4 weeks		Proposal
Too short	29 (22.8%)	7.4 ± 1.9 (6–12)
Appropriate	66 (52.0%)	
Too long	27 (21.3%)	2.2 ± 0.4 (1–3)
Onlay—4 weeks		Proposal
Too short	47 (37.0%)	7.4 ± 1.9 (6–12)
Appropriate	50 (39.4%)	
Too long	7 (5.5%)	1.6 ± 0.7 (1–2)
Complex repair— 4 weeks		Proposal
Too short	60 (47.2%)	7.2 ± 2.3 (5–16)
Appropriate	47 (37.0%)	
Too long	9 (7.1%)	2.3 ± 0.4 (2–3)
Groin hernia repair		
Lichtenstein – 2 weeks		Proposal
Too short	40 (31.5%)	4.5 ± 1.2 (3–8)
Appropriate	71 (55.9%)	
Too long	15 (11.8%)	0.7 ± 0.5 (0–1)
Endoscopic—2 weeks		Proposal
Too short	14 (11.0%)	5.1 ± 3.9 (3–16)
Appropriate	76 (59.8%)	
Too long	36 (28.3%)	0.9 ± 0.4 (0–1)
Abdominal surgery		
Midline laparotomy—4 weeks		Proposal
Too short	40 (31.5%)	7.0 ± 1.6 (6–12)
Appropriate	72 (56.7%)	
Too long	15 (11.8%)	2.1 ± 0.6 (1–3)
Transverse laparotomy—4 weeks		Proposal
Too short	38 (29.9%)	6.6 ± 1.1 (6–10)
Appropriate	67 (52.8%)	
Too long	15 (11.8%)	2.3 ± 0.5 (2–3)
Laparoscopy—2 weeks		Proposal
Too short	6 (4.7%)	4.0 ± 0.0 (−)
Appropriate	73 (57.5%)	
Too long	46 (36.2%)	1.0 ± 0.4 (0–2)

For IPOM, a similar proportion of 52.0% rated 4 weeks appropriate. The remaining rated equally distributed too short or too long. The recommended intervals were about the same as for sublay.

Four weeks were rated appropriate for onlay repair in 39.4%. A large proportion qualified it as too short (37.0%). Only a few rated too long (5.5%). About 18.1% did not rate the proposal for onlay repair.

Most specialists asked (47.2%) considered 4 weeks as too short for complex hernia repair and suggested 5–16 weeks. 37.0% rated appropriate instead; only 7.1% found it to be too long. The differences in the ratings between the surgical procedures were statistically significant (*p* = 0.000, Chi-squared test).

**Fig. 4:**
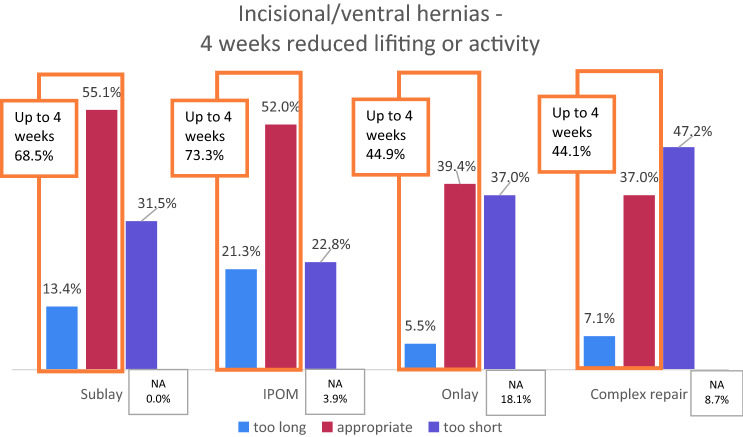
Ratings of 4 weeks reduced physical activity after incisional or ventral hernia surgery. *IPOM* intraperitoneal onlay mesh, *NA* not answered

Put together, 4 weeks of reduced lifting or physical labor was appropriate for the majority after incisional or ventral hernia repair with sublay, IPOM, or onlay. About a third advocated longer intervals of 7 weeks or longer. However, the data also showed that 60–70% considered 4 weeks or shorter for sublay and IPOM reasonable (Fig. 4).

## Discussion

The results of this survey were based on opinions of attending surgeons of the 41st Annual Meeting of the European Hernia Society. We found significant variability in the ratings of the given proposals for restrictions of physical activity. However, our given recommendations were already quite liberal. We saw 60–70% of the participating hernia surgery experts to consider them appropriate or even too long, namely for sublay, IPOM, both open and endoscopic inguinal hernia repair, and midline or transverse laparotomy. The proportion of participants who qualified 2 weeks of reduced activity as too long or appropriate counted 94% for laparoscopy.

Possible reasons to justify restrictive recommendations for convalescence after abdominal or hernia surgery are the risk of recurrence or development of incisional hernias, the fear of causing mesh-related complications, or only pain. Those risks cannot be substantiated by published data and are based mainly on theoretical considerations [10, 11]. For inguinal hernia repair, it has been shown that early and progressive strain or the immediate return to physical work is not associated with hernia recurrence. Consequently, the recommendations for the postoperative recovery to full physical activity have changed and are quite progressive nowadays [8].

### Physical strain and fascial shear stress

One central aspect is the rising intraabdominal pressure associated with lifting. It has been shown that the increase of intraabdominal pressure is dependent on the amount of weight and the way how it is lifted [12]. Moreover, it has been shown that even lifting a weight of 50 kg led to a negligible rise oof the intraabdominal pressure [13]. Of course, physical activity and lifting weights can easily be adapted in the postoperative period, but the effect of these restrictions should be questioned and the impact of lifting weights on intraabdominal pressure and fascial shear stress might be overestimated. Some studies found involuntary actions such as coughing, wheezing, or defecation to cause faster and more significant increases of intraabdominal pressure, which were way higher than that caused by physical activity or lifting [14, 15].

From a biological perspective, the abdominal wall is considered to reach its full strength 3–4 weeks after abdominal surgery [16]. Also, fascial fibroblasts may be activated through physical activity, and fascia might heal faster than the skin incision in turn [17, 18]. If the implantation of a mesh is considered, the physical properties of the operated abdominal wall are further ameliorated. Data from inguinal hernia repair studies emphasized that the abdominal wall is stable on a physiological level immediately after mesh-augmented inguinal hernia repair [6].

A further aspect that stands against the possible negative impact of early strain is the long interval between fascial trauma, i.e., abdominal or hernia surgery, and hernias’ occurrence or recurrence. It has been shown that the majority of incisional hernias occur after 1 year or more (at least 50–60%), and only under 10% developed within 1 year from surgery [5, 19, 20]. Impaired collagen metabolism.

Primary ventral or inguinal hernia development is most likely caused by impaired collagen metabolism [21, 22], which cannot be affected by postoperative resting. Other factors associated with incisional hernia development are the technique of abdominal wall closure, surgical site infections, or comorbidities like obesity or aortic aneurysms [11]. Thus, meshes’ prophylactic implantation after laparotomies is under investigation, as promising results in terms of reduced incisional hernia rates have been shown in high-risk patients [23].

### Evidence for abdominal surgery

In a German hospital surgeons survey, most of the physicians stated they gave recommendations to their patients concerning postoperative strain after abdominal surgery (93% after laparotomies, 77% after laparoscopies) [5]. However, that study reveals 90% of those recommendations were only based on individual or expert opinions rather than available evidence [5].

### Evidence for incisional/ventral hernia surgery

For incisional hernia surgery, Dietz et al. recommended an interval of postoperative reduced activity or lifting restrictions for 3–6 weeks in a recent review [4]. However, they also stated there is no definitive evidence to give binding recommendations. A recently published study found considerable variation in published recommendations on the postoperative strain, a lack of evidence for the guidance given, and, in line with the survey results in this study, substantial variation, and uncertainty of the surgeons, who are supposed to guide their patients [5]. Interesting insights might be expected by a randomized controlled trial that is currently recruiting patients to evaluate the impact of early physical training of the abdominal wall muscles after abdominal surgery on pain, wound healing, and incisional hernia development (https://www.clinicaltrials.gov/ct2/show/NCT03808584#contacts, lastly accessed on November 3, 2020).

### Evidence for groin hernia surgery

The Hernia Surge Study Group published guidelines for inguinal hernia repair. Based on the available evidence, they concluded that there is no need for postoperative activity restrictions after uncomplicated inguinal hernia repair [8]. Moreover, there is no evidence that this would lead to higher inguinal hernia recurrence rates [8]. Consequently, the authors concluded that work or leisure activities could safely be resumed after 3–5 days, both after open or endoscopic inguinal hernia repair [8].

Of course, inguinal hernia repair’s pathophysiological conditions cannot readily be adapted in abdominal surgery or open hernia repair. Based on this study’s findings and the review of the current literature, an early postoperative return to daily life activities in uncomplicated cases might be reasonable and should be further evaluated in prospective design. In our experience, the limit is the individual occurrence of pain (individual full strain). In special conditions, complicated cases or based on an individual therapeutic decision by the surgeon, a substantially longer durations—especially in complex and large hernia repairs—might be recommended.

The resume of full physical strain, sports, and hard work is considered possible after 2 weeks for laparoscopy and inguinal hernia and after 4 weeks for laparotomy and open ventral/incisional hernia repair. In the author’s opinion, recommendations should not exceed 4 weeks after uncomplicated surgery to decrease the risk of unnecessarily long immobilization or sick leave.

### Limitations and controversies

The results are based on expert opinions, which does not take into account the individual confounding factors that are relevant in enhancing or impeding the general wound and tissue repair and healing after any hernia repair (e.g., age, comorbidities, lifestyle and profession, patient’s compliance and insight). That bears an inherent risk of substantial bias. Therefore, the aforementioned statement is only recommended for uncomplicated cases, or after a complication (e.g., a surgical site infection) has already resolved. Also, whether early postoperative return to full activity and strain increases the risk for other complications than hernia development or recurrence (i.e., seroma formation and inflammatory reaction) could be debated and might limit the return to full activity and strain. That needs to be addressed in prospective studies and has to be discussed in that context.

Moreover, it should be noted that there is a lack of well-designed studies, especially for abdominal surgery, ventral or incisional hernia repair and there is a substantial risk for bias as not every patient is seen for follow-up by the initial surgeon. However, we assume usual follow-up rates in hernia surgery to be quite high. In our own experience (Germany), postoperative follow-up rates of at least 70% are necessary to meet quality assurance programs’ requirements in hernia surgery require average follow-up rates of at least 70%. We assume there are similar conditions in other countries (i.e., western Europe/United States). Theoretically, that bears the risk of underestimation of complications, incisional or recurrent hernia development, as the surgeons just are not aware of the true rate of complications. To our knowledge, there is no evidence that patients, who suffer from complications or hernia recurrences, are more prone to be lost to follow-up to their surgeon, that aspect might be relevant for interpreting our results.

The results of this study are based on expert opinions which is only level of evidence D according to GRADE [9]. As evidence-based, validated guidance is needed in clinical routine, recommendations, and convalescence regimes after abdominal and hernia surgery need to be prospectively evaluated.

To conclude, following groin hernia repair (Lichtenstein/endoscopic technique) and laparoscopic operation, the majority agreed on the proposal of 2 weeks refraining from physical strain. Four weeks of no physical strain were considered appropriate by a majority after laparotomy and open incisional hernia repair. However, the results showed substantial variation in the ratings, which indicates uncertainty even in this selected cohort of hernia surgery experts and emphasizes the need for further scientific evaluation. This is particularly remarkable, because a lack of evidence that early postoperative strain leads to higher incisional hernia rates.

## Data Availability

Data tables and analysis results can be requested via email from the corresponding author.
